# TAPBPR isoforms exhibit altered association with MHC class I

**DOI:** 10.1111/imm.12253

**Published:** 2014-04-24

**Authors:** Keith M Porter, Clemens Hermann, James A Traherne, Louise H Boyle

**Affiliations:** Department of Pathology, Cambridge Institute of Medical Research, University of Cambridge, Wellcome TrustCambridge, UK

**Keywords:** antigen presentation/processing alternative splicing, tapasin-related, TAPBPR/TAPBPL

## Abstract

The tapasin-related protein TAPBPR is a novel component of the antigen processing and presentation pathway, which binds to MHC class I coupled with *β*_2_-microglobulin. We describe six alternatively spliced TAPBPR transcripts from the *TAPBPL* gene and investigate three of these at a protein level. TAPBPR transcripts lacking exon 5 result in loss of the membrane proximal IgC domain and loss of ability to bind to MHC class I. Alternative acceptor and donor splice sites in exon 4 of TAPBPR altered the reading frame in the IgV domain and produced a truncated TAPBPR product. An additional exon in the *TAPBPL* gene was identified that encodes extra residues in the cytoplasmic tail of TAPBPR. This longer TAPBPR protein interacted with MHC class I but was attenuated in its ability to down-regulate surface expression of MHC class I. The abundance of these alternative transcripts in peripheral blood mononuclear cells and dendritic cells suggests an important role of TAPBPR isoforms *in vivo*.

## Introduction

Major histocompatibility complex class I molecules are key players in the recognition of tumours and viral infections by the immune system. The MHC class I molecules present peptide antigens at the cell surface to cytotoxic T lymphocytes and their expression is also monitored by natural killer cells. This process is critical for resistance to several infections and it must be tightly regulated. A level of control is performed by the MHC class I antigen processing and presentation pathway, which involves both proteins involved in general glycoprotein folding and specific MHC-encoded components with specialized functions. After folding with the help of calnexin and calreticulin, the MHC class I/*β*_2_-microglobulin (*β*_2_m) heterodimer is incorporated into the peptide loading complex composed of the Transporter associated with antigen processing (TAP) 1 and 2, tapasin, ERp57 and calreticulin (for a recent overview see ref. 1). In this complex, peptides generated from proteasomal degradation are loaded onto the MHC class I heterodimer. Tapasin forms the centrepiece of the peptide loading complex, bridging the MHC class I molecule to the TAP transporter.^2^–^4^ Furthermore, tapasin has been assigned the function of MHC class I peptide optimization, which is important to ensure the selection of stable peptide cargo.^5^–^9^

For the past two decades tapasin was the only known MHC class I dedicated chaperone of the MHC class I antigen processing and presentation pathway. However, we discovered a second MHC class I chaperone; a tapasin-related protein called TAPBPR.^10^ Like its paralogue, tapasin, TAPBPR binds to an MHC class I/β_2_m heterodimer in the endoplasmic reticulum (ER). However, in contrast to tapasin, TAPBPR is not an integral component of the peptide loading complex and its expression is not restricted to the ER.^10^ TAPBPR decreases the rate at which MHC class I molecules mature through the secretory pathway, a role which could be important for peptide selection by MHC class I molecules.

The TAPBPR protein is a type I transmembrane protein consisting of a unique N-terminal domain, an IgV domain and an IgC domain.^11^ Recently, we showed that TAPBPR and tapasin are oriented on MHC class I in a similar manner, using shared amino acids in their IgV and IgC domain to interact with the *α*2-1 and *α*3 domains of MHC class I.^12^ This discovery questions some of the specific roles assigned to tapasin in the MHC class I processing and presentation pathway, such as functions that were discovered by using MHC class I mutants that no longer bound to tapasin. It is apparent now that not only is the function of tapasin lost in these mutants, but also that of TAPBPR, as they share binding sites. In addition, in cells lacking tapasin, the functional effect of TAPBPR on MHC class I is likely to be more prominent, given that MHC class I association with TAPBPR is significantly increased in the absence of tapasin.^12^

The *TAPBPL* gene was proposed to consist of seven exons.^11^ Exons 1 and 2 comprise the TAPBPR signal sequence. Nucleotides from exon 2 and 3 make up the unique N-terminal domain of TAPBPR, exon 4 encodes an IgV domain, while exon 5 encodes the IgC domain. Exon 6 encodes for the transmembrane domain (TMD) of TAPBPR and contributes some residues of the cytoplasmic tail, while exon 7 encodes the cytoplasmic tail.^11^ During our initial cloning of TAPBPR into expression constructs, it became apparent that a number of alternative TAPBPR mRNA products existed in addition to the major TAPBPR transcript. Here we describe some alternatively spliced TAPBPR transcripts at the nucleotide and the encoded protein levels, and investigate the ability of TAPBPR isoforms to associate with MHC class I.

## Materials and methods

### Isolation of peripheral blood mononuclear cells and generation of dendritic cells

Peripheral blood mononuclear cells (PBMCs) were isolated from fresh blood by density gradient centrifugation in Ficoll-Paque as previously described. Human monocytes were selected using *α*CD14 MicroBeads (Miltenyi Biotec, Bergisch Gladbach, Germany, 130-050-201) and used to seed six-well culture plates at a density of 4 × 10^5^ cells per well, before 3-day differentiation using 100 ng/ml granulocyte–macrophage colony-stimulating factor /10% AB serum in a total volume of 1 ml Dulbecco's modified Eagle's medium/well. Immature dendritic cells (DCs) were then either stimulated with 50 U/ml interferon-*γ* (IFN-*γ*) for 72 hr, stimulated with 100 ng/ml lipopolysaccharide for 24 hr, or left unstimulated. The remaining CD14-depleted PBMCs from the initial microbead selection were either harvested immediately for RNA and protein, or stimulated with IFN-*γ* as described above before harvest.

### PCR for alternative TAPBPR transcripts

RNA was extracted from cell lines and primary cells (DCs and PBMCs) using RNEasy™ mini purification kit (Qiagen, Hilden, Germany). Complementary DNA was synthesized from 1 μg of RNA using a QuantiTect™ Reverse Transcription Kit (Qiagen). *TAPBPL* was amplified using specific primers spanning previously reported start and stop codons (Start: 5′-GCAGCCTCCATGGGCACACA-3′, Stop 5′-GGTCAGCTGGGCTGGCTTACA-3′). Amplification was performed using 2·5 U Pfu Turbo DNA polymerase (Stratagene, La Jolla, CA) with 0·5 μm of each primer, 0·2 μm of each dNTP and 1 μl of cDNA in 1× Pfu reaction buffer (Stratagene). The PCR cycling conditions used were as follows: 95° for 3 min then 35 cycles of: 95° for 50 seconds, 68° annealing for 50 seconds and 68° extension for 90 seconds. For each reverse transcriptase product a control reaction was performed using primers for the housekeeping gene *GAPDH* (Forward 5′-CCACCATGGAGAAGGCTGGGGCTCA-3′, Reverse 5′-ATCACGCCACAGTTTCCCGGA-3′) in a total volume of 25 μl containing 1× BioMix Red premix (Bioline, London, UK) and 0·5 μm each primer, and cycled as follows: 96° for 10 min, then 27 cycles of 95° for 50 seconds, 55° for 55 seconds, 72° for 40 seconds. Products were resolved by agarose gel electrophoresis (1·5% agarose/ethidium bromide) and visualized under UV.

### Screening for alternative TAPBPR transcripts

Blunt *TAPBPL* PCR products obtained as above were ligated into pCR®-Blunt II TOPO® (Invitrogen, Carlsbad, CA) as per the manufacturer's instructions. In brief, 4 μl of PCR product was ligated for 5 min at room temperature into 1 μl of vector in the presence of 0·2 m NaCl and 0·01 m MgCl_2_, followed by transformation into One Shot® TOP10 Chemically Competent *Escherichia coli* and selection on Kanamycin^+^ LB agar plates. Colonies containing ligation products were identified by PCR using vector-specific primers and size discrimination by gel electrophoresis. In brief, colonies were picked into 100 μl LB Broth/kanamycin and incubated at 37° for 2 hr, followed by amplification of 2 μl of colony supernatant with vector-specific SP6 primer (5′-ATTTAGGTGACACTATAG-3′) and T7 primer (5′-TAATACGACTCACTATAGGG-3′) in a total volume of 25 μl containing 1× BioMix Red premix (BIO-25006, Bioline) and 0·5 μm each primer, and cycled as follows: 96° for 10 min, then five cycles of 95° for 24 seconds, 71° for 45 seconds, 72° for 30 seconds, followed by 32 cycles of 96° for 25 seconds, 68° for 45 seconds, 72° for 30 seconds, then five cycles of 96° for 25 seconds, 55° for 1 min, 72° for 2 min and one final extension at 72° for 10 min. Products were resolved by agarose gel electrophoresis (1·5% agarose/ethidium bromide) and clones containing inserts of an appropriate size were subsequently sequenced.

### Expression of TAPBPR isoforms in HeLa cells

Inserts of selected alternative TAPBPR transcripts cloned into pCR®-Blunt II TOPO® vectors were further subcloned into the lentiviral expression vector pHRSIN-C56W-UbEM. These were transfected into HEK-293T cells using TransIT-293 (Mirus, Madison, WI), together with the pCMVR8.91 packaging vector and pMD-G envelop vector. Supernatants collected approximately 48 hr and 72 hr post-transfection were used to transduce HeLa-M cells. This results in the expression of TAPBPR under the spleen focus-forming virus promoter and the green fluorescent protein (GFP) derivative Emerald under the ubiquitin promoter, which serves as a reporter for transduction efficiency.

### Immunoprecipitation and Western blot analysis

To detect whether any TAPBPR protein products were secreted, HeLa cells stably transduced with the TAPBPR variants were incubated at 37° overnight in Opti-MEM serum-free media. The cell culture supernatant was collected, centrifuged to remove any cells, and proteins were precipitated using phenol–chloroform extraction. Whole cell lysates were made in Tris-buffered saline (TBS; 20 mm Tris–HCl, 150 mm NaCl, 5 mm MgCl_2_, 1 mm EDTA) containing 10 mm *N*-ethylmaleimide (Sigma, St Louis, MO), 1 mm PMSF and protease inhibitors (Roche, Burgess Hill, UK) containing either 1% digitonin (Merck Millipore, Billerica, MA) or 1% Triton X-100 (Sigma) at 4°. Post-nuclear lysates were precleared on IgG-and protein A-Sepharose beads (GE Healthcare, Little Chalfont, UK). Immunoprecipitations were performed for TAPBPR using either the rabbit polyclonal antisera R039 or the mouse monoclonal antibody (mAb) PeTe4 both raised against the extracellular portion of human TAPBPR^10^ as indicated together with protein A-sepharose at 4°. Beads were washed thoroughly in 0·1% detergent in TBS. Samples were heated at 80° for 10 min in sample buffer with 100 nm *β*-mercaptoethanol before separation by SDS–PAGE. Proteins were transferred onto an Immobolin transfer membrane (Merck Millipore) then blocked in 5% (weight/volume) dried milk in 0·1% Tween-20–PBS. Membranes were incubated with either the MHC class I heavy specific mAb HC10, mouse anti-TAPBPR ab57411, (Abcam, Cambridge, UK), or rabbit anti-calnexin (Enzo Life Sciences, Exeter, UK) followed by species-specific secondary antibodies (Dako, Glostrup, Denmark) and detection by enhanced chemiluminescence (GE Healthcare).

### Pulse–chase analysis

Harvested cells were washed in PBS then incubated in cysteine/methionine-free RPMI-1640 supplemented with 5% dialysed fetal calf serum, 2 mm glutamine and 20 mm HEPES for 30 min at 37°. Cells were then labelled with [^35^S]methionine/cysteine Promix (Amersham Pharmacia) for 10 min at 37° and subsequently chased for 0–6 hr in RPMI containing 10% fetal calf serum supplemented with 3 mm unlabelled methionine and 60 μm unlabelled cysteine. After lysis in 1% digitoin TBS, TAPBPR was immunoprecipitated from pre-cleared post-nuclear lysates using the TAPBPR-specific mAb PeTe4 and protein A–sepharose. After extensive washing in 0·1% digitonin TBS, samples were heated at 80° for 10 min in sample buffer with 100 nm *β*-mercaptoethanol followed by digestion with 1000 U of endoglycosidase H_f_ (Endo H_f_; NEB, Ipswich, MA) for 1 hr at 37°. After separation by SDS–PAGE, gels were fixed in 40% methanol, 12% acetic acid, dried and images were obtained on a phosphor screen (Perkin-Elmer, Waltham, MA). PhorphorImager analysis was performed using a Typhoon Trio variable mode imager (GE Healthcare) with imagequanttl software.

### Flow cytometry

HeLa and HeLa-expressing TAPBPR variants were detached from flasks using trypsin. To allow membrane recovery post-trypsination the cells were resuspended in RPMI supplemented with 10% fetal calf serum for 30 min at 37°, followed by incubation at 4°. Cells were stained with an anti-HLA-A68 specific mAb (One Lambda, Canoga Park, CA) or with the TAPBPR-specific mAb PeTe4 for 30 min at 4°. Cells were stained with a mouse IgG2a as a negative control (Dako). Following extensive washing in PBS, cells were stained with AlexaFluor 647 goat anti-mouse IgG (Life Technologies, Paisley, UK) for 30 min at 4°. Cells were analysed on a BD Bioscience FACS Calibur 4-colour analyser.

## Results

### Alternative TAPBPR transcripts encoded by exon splicing

**Figure 1:**
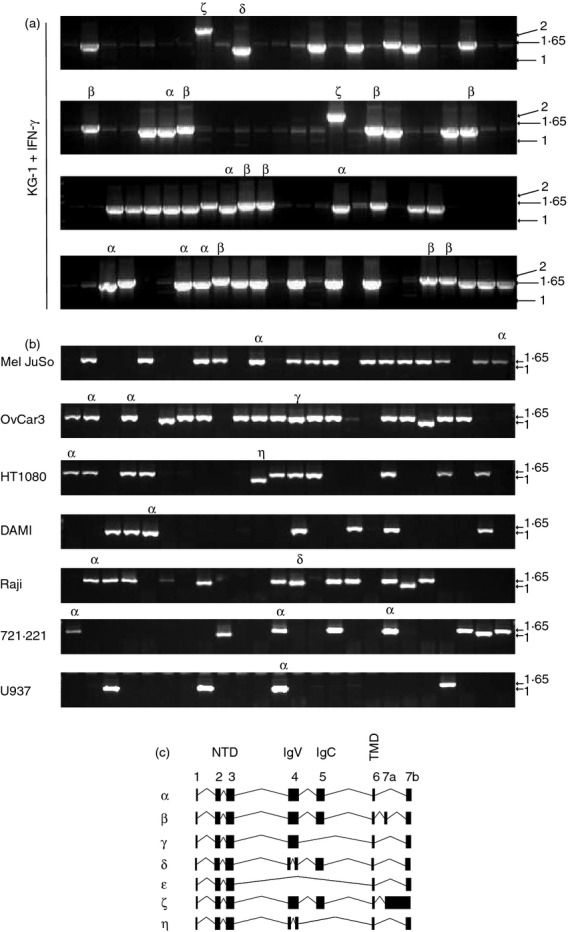
Alternative TAPBPR transcripts. (a, b) TAPBPR was amplified from cDNA generated from the indicated cell lines using primers specific for exon 1 and exon 7 of TAPBPR. The PCR products were cloned into the pCR-blunt-II TOPO vector and colonies containing inserts were identified by PCR using primers Sp6 and T7. A selection of colonies was further screened by sequence analysis. PCR products identified as alternative TAPBPR isoforms are highlighted with a symbol on the PCR screen. (c) Exon structure of seven human TAPBPR transcripts. cDNA sequences were aligned against human *TAPBPL* gene on chromosome 12p13.3 (OMIM:607081) using spidey mRNA genomic alignment software (http://www.ncbi.nlm.nih.gov/spidey/) using default settings. The transcript for TAPBPR *α* has previously been reported (NM_018009.4). *β*,*γ*,*δ*,*ε*,*ζ*,*η* mRNA products have not been described previously.

During the initial process of cloning TAPBPR into expression constructs, it became apparent that a number of alternative TAPBPR mRNA products existed in addition to the major TAPBPR transcript.^10^ We investigated the alternative transcripts using PCR, followed by cloning individual TAPBPR transcripts into the pCR®-Blunt II TOPO vector and screening colonies by sequence analysis. Using primers specific for TAPBPR exon 1 and exon 7 we amplified the major TAPBPR coding sequence, the *α* transcript, which was found in all cell types tested (Fig. 1 and Table 1). In IFN-*γ*-treated KG-1 cells we identified a longer TAPBPR mRNA product that we named the TAPBPR *β* transcript (Fig. 1a). Although the *TAPBPL* gene is thought to be composed of seven exons,^11^ the TAPBPR *β* transcript has an additional exon after exon 6, which we refer to as exon 7a.

**Table 1:** Cell lines in which alternative transcripts are found

Transcript	Source identified from	Detection in primary cells
*α*	All cell lines tested	all tissues tested, PBMCs, DCs
*β*	KG-1	PBMCs, DCs
*γ*	THP-1, 721.221, Ovcar	spleen, PBMCs, DCs
*δ*	KG-1, THP-1	PBMCs, DCs
*ε*	THP-1	
*ζ*	KG-1	
*η*	HT1080	
*θ*	Raji	

DCs, dendritic cells; PBMCs, peripheral blood mononuclear cells.

Other variously sized PCR products were also detected in KG-1 and the other cell lines (Fig. 1). Sequencing of these products revealed a number of alternative TAPBPR transcripts. Numerous mRNA products were also isolated that were shorter than the TAPBPR *α* transcript. We isolated a transcript, which we named TAPBPR *γ* transcript, in which exon 5 was missing. This transcript was identified on OvCar3 cells (Fig. 1b) as well as in THP-1 and 721.221 cells (Table 1). Furthermore, in THP-1 cells we found a TAPBPR transcript in which both exon 4 and exon 5 were spliced out, which we named TAPBPR *ε* (Table 1 and Fig. 1c). There also appears to be an additional acceptor and donor splice site in exon 4 of TAPBPR since we isolated TAPBPR transcripts which had a ‘bridged’ exon 4. The TAPBPR *δ* transcript contained this ‘bridged exon 4’ while retaining all other TAPBPR exons (Fig. 1 and Table 1). This ‘bridged exon 4’ was also identified in other transcripts including the *η* transcript in which exon 5 is additionally spliced out (Fig. 1, Table 1, and see Supporting information, Fig. S1). One additional TAPBPR transcript described here is the *ζ* transcript, which contains exon 7a and retains the intron sequence between exon 7a and 7b. This transcript was only identified in KG-1 cells (Fig. 1 and Table 1). For the nucleotide sequence of the various splice forms see Fig. S1.

Although some alternative TAPBPR transcripts have already been deposited for the human *TAPBPL* gene (see http://www.ensembl.org – TAPBPL ENSG00000139192), none of the specific TAPBPR isoforms described here have so far been described, apart from the TAPBPR *α* transcript. However, both the previously deposited sequences and the alternative transcripts identified here share some features in common such as the existence of the bridged exon 4 in the *TAPBPL* gene (see Supporting information, Fig. S2).

### Alternative TAPBPR transcripts are expressed in primary cells

**Figure 2:**
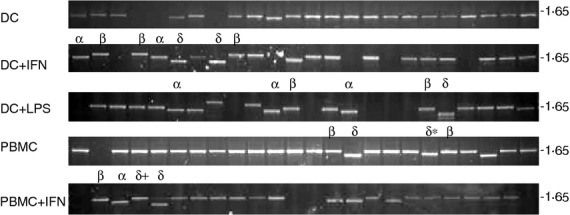
Detection of alternative TAPBPR transcript in peripheral blood mononuclear cells (PBMCs) and dendritic cells (DCs). TAPBPR was amplified from cDNA generated from PBMCs and DCs treated as indicated using primers specific for exon 1 and exon7 of TAPBPR. The PCR products were cloned into the pCR-blunt-II TOPO vector and colonies containing inserts were identified by PCR using primers Sp6 and T7. A selection of colonies was further screened by sequence analysis. Sequenced inserts are highlighted with a symbol above indicating the TAPBPR transcript identified. *δ*^+^ contains bridged exon 4 and an alternative exon 7b which at a protein levels looks identical to delta. δ* contains bridged exon 4 and exon 7b which at a protein level looks identical to *δ*.

So far we have described the existence of alternative TAPBPR transcripts in human cell lines. However, we wanted to determine if these alternative transcripts were expressed *in vivo*. To this end, we examined the presence of alternative TAPBPR transcripts in PBMC and DC, using our original method of cloning individual TAPBPR transcripts into the pCR®-Blunt II TOPO vector and screening colonies by sequence analysis. Via this procedure, we confirmed the abundant expression of TAPBPR *α*,*β* and *δ* transcripts in PBMCs and DCs (Fig. 2). *In vivo* expression of TAPBPR *β* and TAPBPR *γ* was furthermore confirmed in a human tissue panel using a transcript-specific PCR readout (see Supporting information, Fig. S3).

### TAPBPR protein prediction

**Figure 3:**
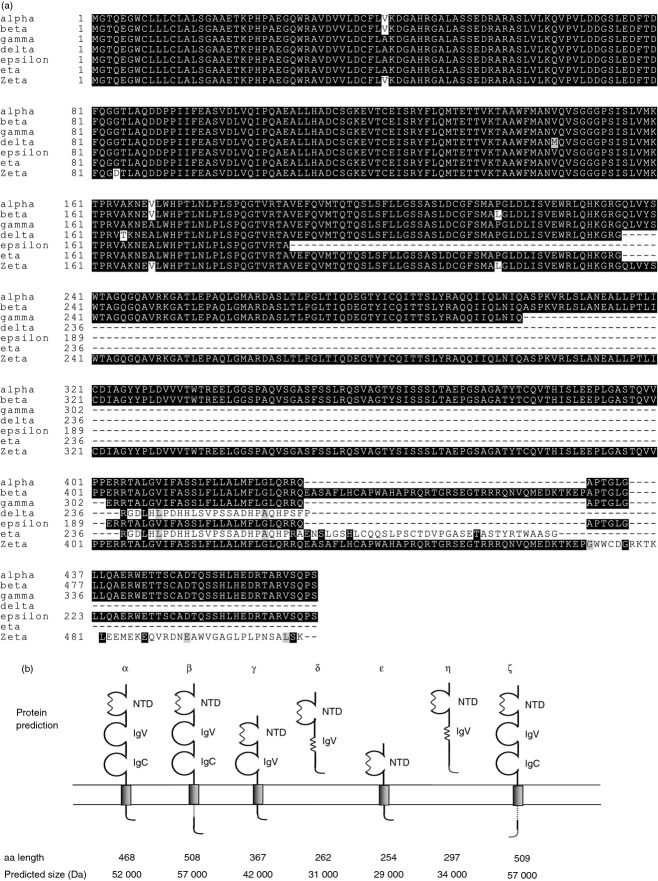
Predicted protein products from the seven alternative TAPBPR isoforms. The sequenced PCR products for the seven alternative TAPBPR isoforms were translated. (a) Alignment of the amino acids encoded from the alternative TAPBPR transcripts. The sequences were aligned using clustal w and the figure was generated using BoxShade. (b) Schematic representations of the predicted TAPBPR isoforms. Amino acid length and predicted molecular weight are shown below each prediction.

The TAPBPR *α* transcript encodes a protein consisting of three luminal domains, a TMD and cytoplasmic tail (Fig. 3). The TAPBPR *β* protein is predicted to have the same luminal domain as TAPBPR *α*; however, the extra exon (7a) is predicted to encode additional amino acid residues EASAFLHCAPWAHAPRQRTGRSEGTRRRQNVQMEDKTKEP in the cytoplasmic tail of this TAPBPR isoform (Fig. 3). TAPBPR *ζ* also has the same luminal domains as TAPBPR *α*; however, the retention of the intron between exon 7a and 7b alters the reading frame after exon 7a and produces an alternative end to the cytoplasmic tail (Fig. 3). TAPBPR *γ*, in which exon 5 is spliced out, encodes a protein lacking the IgC domain. However, the transcript remains in frame with exon 6 and therefore is predicted to have an intact TMD and cytoplasmic tail. TAPBPR *ε*, in which exons 4 and 5 are spliced out, encodes a protein product that is predicted to lack both the IgV and IgC domains but again still has an intact TMD and cytoplasmic tail coming directly after the unique N-terminal domain (Fig. 3). TAPBPR *δ* with the ‘bridged’ exon 4 is predicted to encode a protein with the unique N-terminal domain of TAPBPR and part of the IgV domain. However, the additional acceptor and donor splice site in exon 4 changes the reading frame of the mRNA and is predicted to result in a truncated protein because of a stop codon in exon 5. Similarly TAPBPR *η* with its bridged exon 4 and spliced out exon 5 is also predicted to encode a protein consisting of the unique N-terminal domain and partial IgV domain. Again there is a change of reading frame producing a protein that lacks a predicted TMD (Fig. 3).

### TAPBPR protein expression from the alternative TAPBPR transcripts

**Figure 4:**
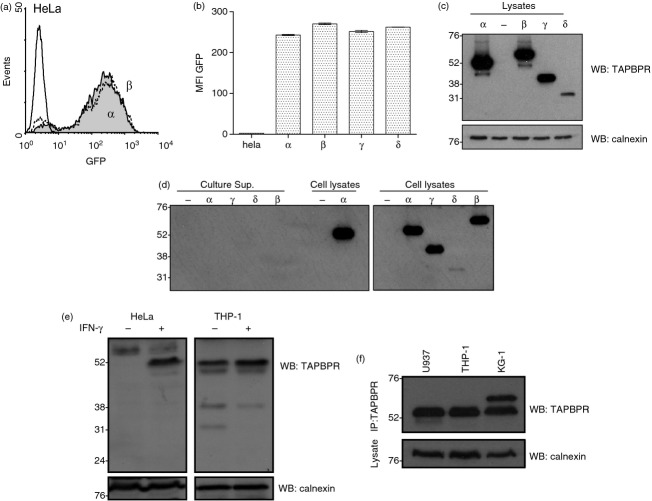
Protein translation from the alternative TAPBPR transcripts. (a) Cytofluorometric analysis for green fluorescent protein (GFP) expression from HeLa cells (black line histogram) and HeLa transduced with a lentiviral expression vector with a bi-cistronic GFP reporter and either TAPBPR *α* (grey-filled histogram) or TAPBPR *β* (black dotted line). (b) Bar graph of mean fluorescence intensity (MFI) of GFP on the full panel of TAPBPR isoforms from three independent experiments (Error bars: ± SEM). (c) Western blot analysis for TAPBPR (using mouse anti-TAPBPR; Abcam) or calnexin as a loading control from non-transduced HeLa (−) or HeLa stably transduced with lentiviral expression construct containing TAPBPR *α*,*β*,*γ*,*δ*. (d) Western blot analysis for TAPBPR from precipitated culture supernatants of non-transduced HeLa (−) or HeLa stably tranduced with TAPBPR *α*,*β*,*γ* or *δ* expression constructs. Western blot for TAPBPR on cellular lysates from the same cells is included as a positive control. (e) Western blot analysis for TAPBPR (using mouse anti-TAPBPR; Abcam) or calnexin as a loading control on HeLa and THP-1 cells ± IFN-*γ* treatment. (f) TAPBPR was isolated by immunoprecipitation (using polyclonal antiserum R039) from U937, THP-1 and KG-1 cells. Western blot analysis was performed for TAPBPR using mouse anti-TAPBPR. Western blotting with calnexin on lysates is included as a loading control.

We chose to investigate in-depth the three most abundant alternative TAPBPR transcripts, which were found multiple times in a number of different cell lines (Table 1). We focused on TAPBPR *β*,*γ* and *δ*, comparing various properties of these alternative transcripts with TAPBPR *α*. First, to determine if the three alternative TAPBPR transcripts produced an alternative TAPBPR protein product, we cloned cDNA from the TAPBPR *β*,*γ*,*δ*, transcripts into lentiviral expression vectors containing a bi-cistronic GFP reporter. The constructs were then transduced into HeLa cells, which do not express TAPBPR unless treated with IFN-*γ*.^10^ Flow cytometry for the GFP reporter revealed good transduction efficiency for all TAPBPR containing vectors into HeLa (*α* = 93%, *β* = 88%, *γ* = 93%, *δ* = 91%) (*α* and *β* are shown in Fig. 4a). The mean fluorescence intensity of GFP was comparable in all cell lines ranging from mean fluorescence intensity of 243 for TAPBPR *α* transduced cells to an mean fluorescence intensity of 270 for TAPBPR *β* transduced cells (Fig. 4b). To detect transduced TAPBPR protein, Western blotting was performed for TAPBPR using an antibody raised against amino acid 23–122 of TAPBPR, a region encoded in all of the TAPBPR isoforms. This revealed significant protein expression of TAPBPR *α*,*β* and *γ* (Fig. 4c). However, we failed to detect significant levels of the TAPBPR *δ* protein when expressed in HeLa although a faint band was observed in Western blot experiments (Fig. 4c). As TAPBPR *δ* does not contain a TMD, one reasonable explanation for the lack of significant detection in cell lysates by Western blot analysis was that this protein product was secreted by the HeLa cells. To determine if this was the case, HeLa cells transfected with the various isoforms of TAPBPR were cultured overnight in serum-free media. This cell culture medium was then collected, concentrated, separated by SDS–PAGE, and then Western blotting for TAPBPR was performed. However, we failed to detect any secreted TAPBPR *δ*, or any of the other TAPBPR isoforms, in the cell culture supernatants (Fig. 4d). Therefore, the lack of detection of TAPBPR in cells does not appear to be the result of protein secretion. As the stop codon encoded in TAPBPR *δ* is more than 50 nucleotides upstream of the 3′ exon–exon junction, it is likely that this transcript is subject to nonsense-mediated mRNA decay and so is not translated into a protein product in any significant quantities.^13^,^14^

### Evidence for endogenous protein expression

Next we screened the cell lines for the alternative TAPBPR protein expression. The TAPBPR *α* transcript was abundantly expressed in THP-1, U937, KG-1 and IFN-*γ*-treated HeLa cells (Fig. 4e,f). In KG-1 cells, an additional higher molecular weight TAPBPR protein product with the predicted size of the TAPBPR *β* transcript was detected (Fig. 4f). This larger protein product is definitely TAPBPR because it was detected in TAPBPR immunoprecipitation–Western blot experiments. In cell lines such as THP-1 smaller TAPBPR protein products, the predicted size of TAPBPR *γ* and *δ*/*ε*, were detected (Fig. 4e). Taken together, these data suggest that some of the alternative TAPBPR transcripts produced endogenously within cells are translated into alternative TAPBPR proteins.

### TAPBPR *γ* does not interact with MHC class I

**Figure 5:**
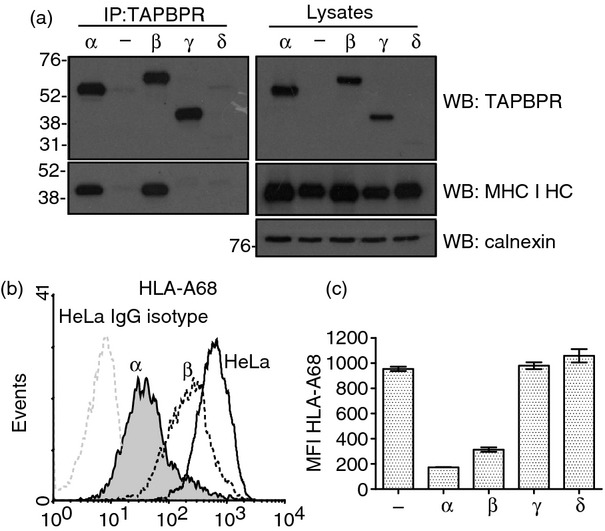
Association of the TAPBPR isoforms with MHC class I. (a) TAPBPR was isolated by immunoprecipitation (using polyclonal antiserum R039) from the panel of HeLa cells stably transduced with the TAPBPR isoforms lysed in 1% Triton X-100 Tris-buffered saline. As a negative control non-transfected HeLa cells were included (−). Western blot analysis was performed for TAPBPR using mouse anti-TAPBPR, the MHC class I heavy chain using HC10 or calnexin on TAPBPR immunoprecipitated or lysates as indicated. (b) Cytofluorometric analysis for surface HLA-A68 expression from HeLa cell (black line histogram) and HeLa transduced with TAPBPR *α* (grey-filled histogram) or TAPBPR *β* (black dotted line). Staining of non-transduced HeLa with an isotype control is included as a negative control (grey-dotted histogram). (c) Bar graph of mean fluorescence intensity (MFI) of surface HLA-A68 on the full panel of cells expression the TAPBPR isoforms from two independent experiments (Error bars: ± SEM).

The cellular ligand for TAPBPR is the MHC class I/β_2_m heterodimer. Next we determined if the alternative TAPBPR isoforms are still capable of interacting with MHC class I. TAPBPR was immunoprecipitated from HeLa cells expressing the various TAPBPR isoforms, followed by Western blotting for MHC class I. As expected, a strong association was detected between TAPBPR and MHC class I for TAPBPR *α* protein (Fig. 5a). In addition, the TAPBPR *β* protein with the longer cytoplasmic tail also associated strongly with MHC class I (Fig. 5a). However, the TAPBPR γ protein, which lacks the IgC domain, did not associate with MHC class I (Fig. 5a).

### TAPBPR *β* does not down-regulate MHC class I surface expression as efficiently as TAPBPR *α*

The over-expression of TAPBPR *α* in HeLa cells reduced surface expression of HLA-A68 to under 20% of that on non-transduced HeLa.^10^,^12^ Next we wanted to determine if the TAPBPR *β* protein, which strongly associated with MHC class I, exhibited the same functional effect on HLA-A68 surface expression as the TAPBPR *α*. In agreement with our previously published findings the expression of TAPBPR *α* resulted in a significant down-regulation of HLA-A68 from the cell surface (Fig. 5b,c). However, when TAPBPR *β* was expressed at the same level as TAPBPR *α*, it had a limited effect, reducing surface expression of HLA-A to 33% of that on non-transduced HeLa cells (Fig. 5b,c). This is intriguing as the MHC class I binding site is clearly intact in TAPBPR *β*, with the only alterations to residues in its cytoplasmic tail. In contrast, the over-expression of TAPBPR *γ* and *δ*, which did not associate with MHC class I in HeLa, did not alter cell surface expression of HLA-A68 (Fig. 5c).

### TAPBPR *β* exhibits altered trafficking compared with TAPBPR *α*

**Figure 6:**
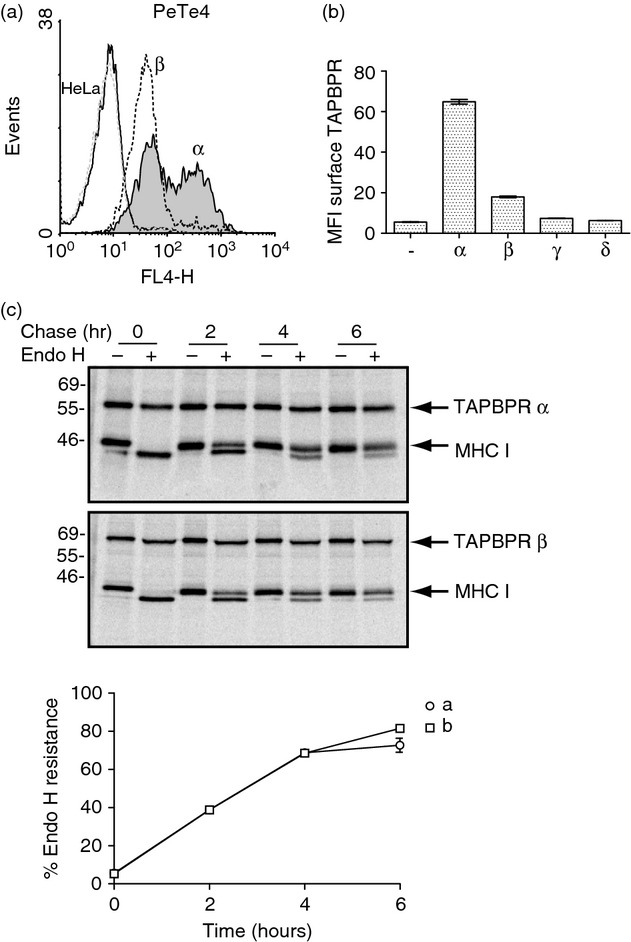
Altered trafficking of TAPBPR *β*. (a) Cytofluorometric analysis for surface TAPBPR expression on HeLa cells (black line histogram) and HeLa transduced with TAPBPR *α* (grey-filled histogram) or TAPBPR *β* (black dotted line). Staining of non-transduced HeLa cells with an isotype control is included as a negative control (grey-dotted histogram). (b) Bar graph of mean fluorescence intensity (MFI) of surface TAPBPR on the full panel of cells expression the TAPBPR isoforms from two independent experiments (Error bars: ± SEM). (c) The anterograde transport rate of TAPBPR *β* was compared with TAPBPR *α* expressed in HeLa cells using pulse–chase analysis. HeLa cells expressing TAPBPR *α* and TAPBPR *β* were labelled with [^35^S]methionine/cysteine for 30 min and chased for 0–6 hr as indicated. Following lysis in 1% digitonin Tris-buffered saline, TAPBPR was isolated from pre-cleared post-nuclear supernatants by immunoprecipitation using the mouse monoclonal antibody PeTe4. Following elution and denaturation the samples were treated with or without endoglycosidase H (Endo H). The signal intensity of the MHC class I heavy chain band was determined by densitometry and the amount of Endo-H-resistant MHC class I associated with TAPBPR at each time-point was plotted as a percentage of the Endo-H-resistant MHC class I associated with TAPBPR at the 0 time-point.

As the cytoplasmic tails of type I transmembrane proteins often contain trafficking motifs that control cellular localization, we determined if TAPBPR *β* with its altered cytoplasmic tail trafficked differently to TAPBPR *α*. TAPBPR *α* is not restricted to the ER and is exported through the medial Golgi.^10^ Although we failed to detect any significant cell surface expression of endogenous expressed TAPBPR by flow cytometry, the over-expression of TAPBPR *α* in HeLa cells resulted in a significant proportion being expressed at the cell surface (Fig. 6a,b and ref. 10 Fig S4.). Two populations of surface expressed TAPBPR, high and intermediate, were consistently observed by flow cytometry upon TAPBPR *α* transduction in HeLa. However, when TAPBPR *β* was over-expressed in HeLa, the population with the high level of expression was not observed (Fig. 6a). Hence, the additional residues in the cytoplasmic tail appeared to alter trafficking of TAPBPR. In contrast to TAPBPR *α* and *β*, over-expression of TAPBPR *γ* and *δ* did not result in any significant cell surface expression (Fig. 6b).

The reduced cell surface expression of TAPBPR *β* could be the result of a reduction in the rate of ER export of TAPBPR *β* compared with TAPBPR *α*. To investigate this, pulse–chase analysis followed by digestion with Endo H was performed. As human TAPBPR contains no N-linked glycosylation sites, we examined the Endo H sensitivity of MHC class I associated with TAPBPR as a surrogate. This failed to reveal any alteration in the anterograde transport rate of TAPBPR *β* compared with TAPBPR *α* (Fig. 6c). Therefore the alteration in trafficking of TAPBPR *β* appears to occur after the medial Golgi.

## Discussion

TAPBPR is a novel component of the MHC class I antigen processing and presentation pathway. Like tapasin, TAPBPR binds to a heterodimer of MHC class I heavy chain/*β*_2_m in the ER.^10^ In contrast to tapasin, TAPBPR is not an integral component of the peptide loading complex and its expression is not restricted to the ER.^10^ While cloning the dominant TAPBPR *α* transcript, we isolated a number of alternative TAPBPR splice products. Here we have described six alternative TAPBPR transcripts that were easily detected using our PCR/cloning based method. However, it is highly likely that other transcripts also exist.

Although TAPBPR was originally thought to be composed of seven exons,^11^ it is now apparent that an eighth exon also contributes to the coding region of the TAPBPR cytoplasmic tail with the discovery of the TAPBPR *β* transcript. Therefore, the coding DNA sequence of both tapasin and TAPBPR genes comprises eight exons with the TMD/cytoplasmic tail of both proteins being encoded by three exons. Furthermore, in addition to conventional exon skipping contributing to the production of TAPBPR isoforms, we found that exon 4 of TAPBPR has an internal acceptor : donor site which splices out part of the IgV domain. In the two transcripts containing the ‘bridged’ exon 4 isolated so far, the reading frame is changed, producing a truncated TAPBPR protein.

The ability of the *TAPBPL* gene to produce alternative splice products is not specific to humans, as alternatively spliced TAPBPR transcripts have also been found in rainbow trout.^15^ Two TAPBPR splice products have been reported in fish: one lacking the nucleotides that encode the IgC domain and another lacking the nucleotides encoding the transmembrane domain and cytoplasmic tail. Although some of the human TAPBPR transcripts identified here were only found in a single cell line (such as TAPBPR *ε*,*η*,*θ*) and are therefore likely to have limited expression, the TAPBPR *β* and *δ* transcripts were more abundant. Of particular note is the abundance of the TAPBPR *β* transcript in DCs and PBMCs. This high abundance of the TAPBPR *β* transcript was also observed in KG-1 cells and resulted in TAPBPR *β* protein being expressed at equal levels as TAPBPR *α*.

In contrast to frequent detection of alternative TAPBPR splice product from the *TAPBPL* gene, alternative transcripts from the tapasin gene are less abundant. A tapasin transcript lacking exon 2 has been identified in the tapasin defective cell line 721.220.^16^ This is caused by a single nucleotide substitution which disrupts the 5′ splice site of the second intron and produces a protein with a truncated signal sequence and lacks the first 49 amino acids of the tapasin N-terminal domains.^16^ In addition, an alternatively spliced tapasin transcript containing intron 5 has also been described.^17^ This introduces a new stop codon, producing a tapasin molecule composed of the luminal domains but lacking a TMD and conventional cytoplasmic tail. Furthermore, a tapasin transcript lacking exon 3 has been identified in a human melanoma cell line and in cells infected with human cytomegalovirus.^18^,^19^ This deletes amino acids 70–156 of tapasin, eliminating the central *β* sheet of the N-terminal three-tiered *β* sheet sandwich, which is essential for tapasin to interact with ERp57. Although alternative tapasin transcripts can be detected in normal cells, they are significantly less abundant than the wild-type tapasin transcript and may only contribute to the MHC class I pathway in situations in which full-length tapasin is lost.

The production of alternative protein isoforms can alter the expression level of protein, abolish protein–ligand interactions, change the subcellular localization of a protein, and regulate protein function by acting as a dominant negative (for a recent review see ref. 20). With the TAPBPR protein isoforms characterized here we found that protein–ligand interaction was altered for TAPBPR *γ*. This isoform lacking the IgC domain was unable to associate with MHC class I. This is consistent with our finding that residues R335 and Q336/S33 in the IgC domain of TAPBPR are crucial for its association with MHC class I.^12^ Subcellular localization of TAPBPR also appears to be affected by splicing events. For the TAPBPR *β* isoform, with its extended cytoplasmic tail, we observed lower cell surface expression compared with the TAPBPR *α* isoform. This was not due to ER retention, as the rate of anterograde transport of TAPBPR *β* was similar to TAPBPR *α*. Although we know endogenously expressed TAPBPR *α* exports through the medial-Golgi with MHC class I, we are currently unsure of the final subcellular destination of endogenously expressed TAPBPR after the Golgi.^10^ However, the fact that different isoforms of TAPBPR show alteration in localization beyond the Golgi suggests that this post-Golgi pool of TAPBPR is important regarding TAPBPR's function in relation to MHC class I. This is further highlighted by the fact that over-expression of TAPBPR *β* does not down-regulate cell surface expression of MHC class I as efficiently as TAPBPR *α*. Since TAPBPR *β* is predominately expressed in cells which can cross-present, such as DCs and KG-1 cells,^21^–^25^ an intriguing speculation is that TAPBPR *β* might play a role in the MHC class I cross-presentation pathway. We are currently focusing on the specific function of major TAPBPR *α* isoform. Once this has been determined detailed functional characteristics of these alternative TAPBPR isoforms can be investigated.

## Supporting Information

Figure S1 Nucleotide sequence of TAPBPR transcripts.

Figure S2 Alternative TAPBPR transcripts previously deposited for the human *TAPBPL* gene.

Figure S3 Expression of the TAPBPR *β* and *γ* transcripts in human tissue.
