# Supplementary figures and images for: TAPBPR isoforms exhibit altered association with MHC class I
Source: Immunology. 2014 Apr 24;142(2):289–99. doi: 10.1111/imm.12253 (PMC4008236; doi:10.1111/imm.12253)

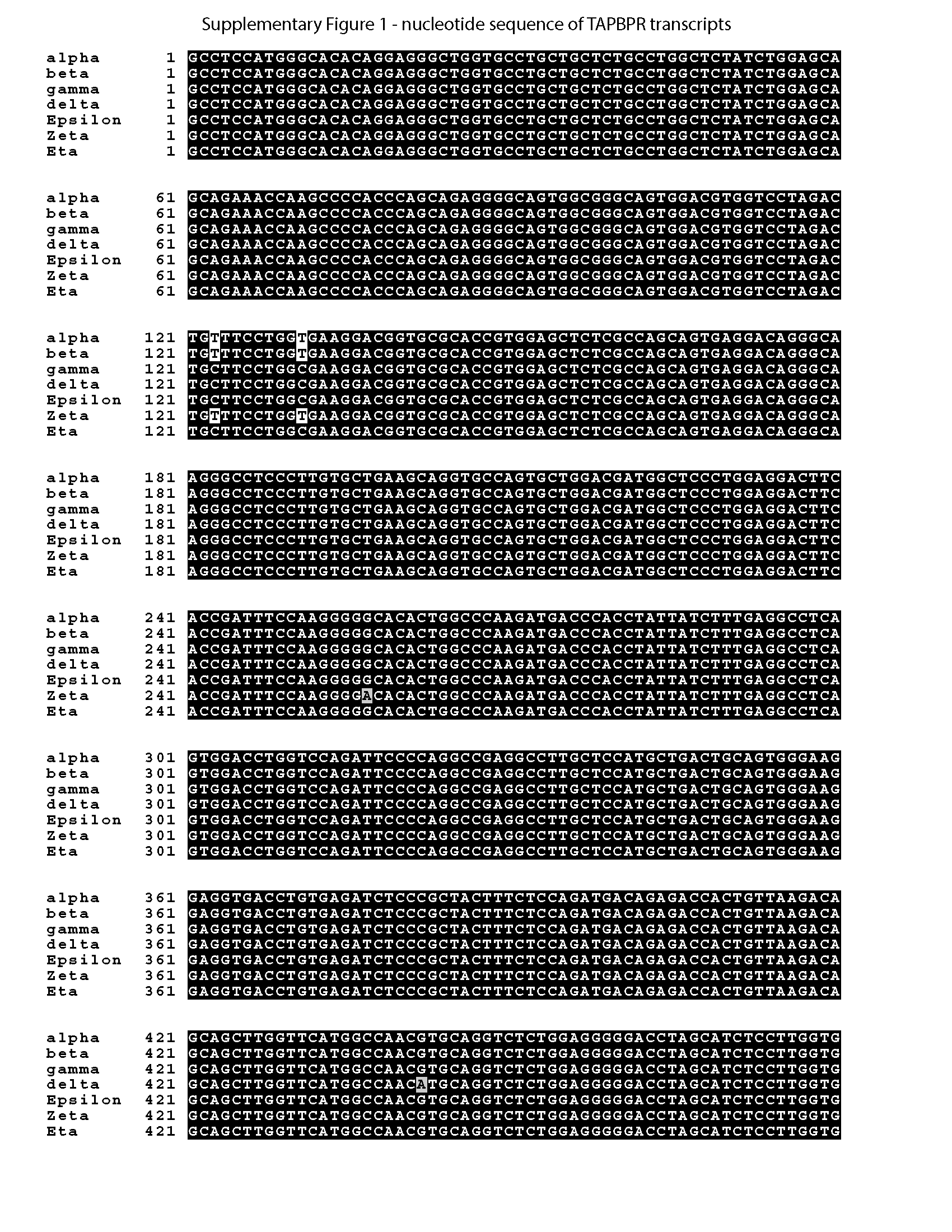

Supplement: Figure S1 — Nucleotide sequence of TAPBPR transcripts. [file imm0142-0289-sd1.tiff]

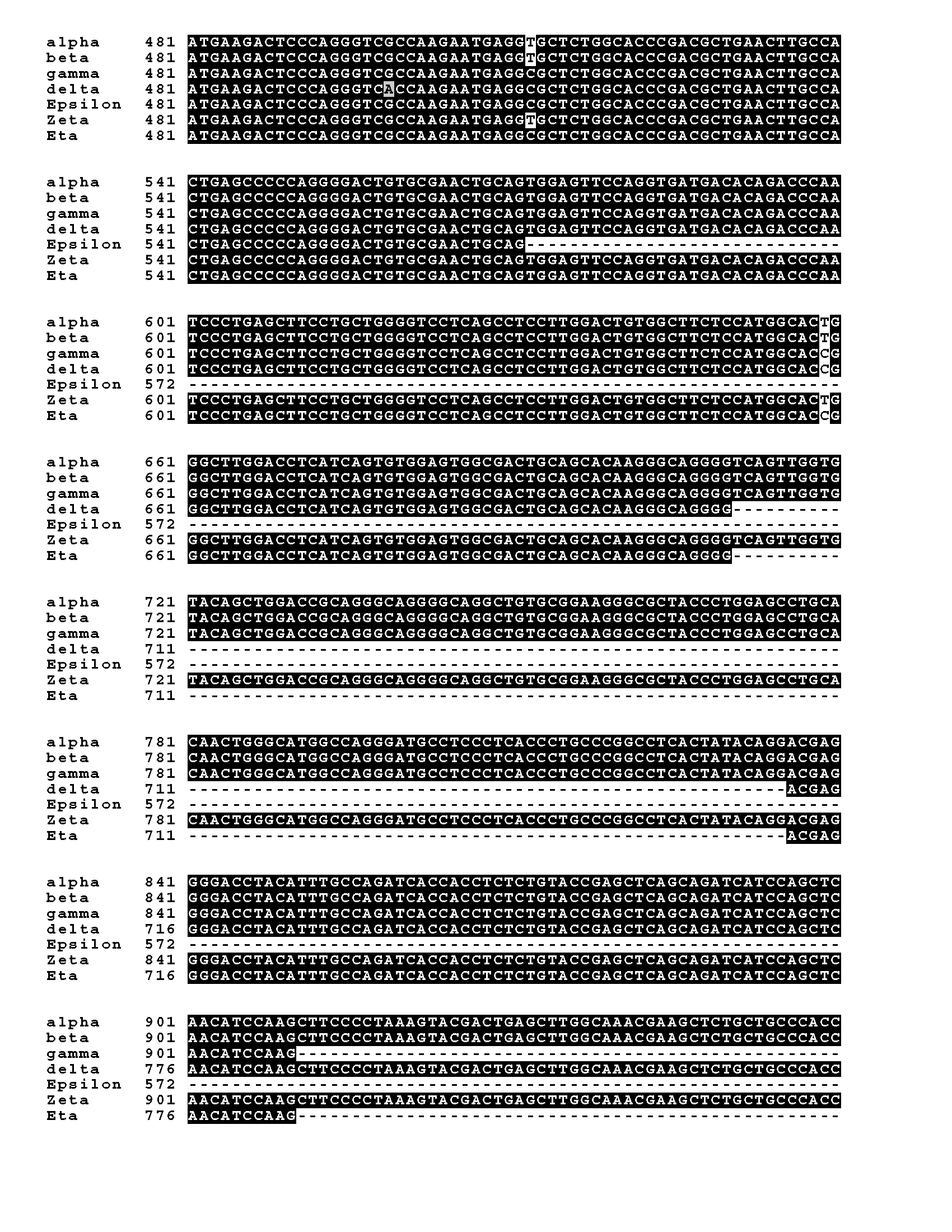

Supplement: Figure S2 — Alternative TAPBPR transcripts previously deposited for the human TAPBPL gene. [file imm0142-0289-sd2.tiff]

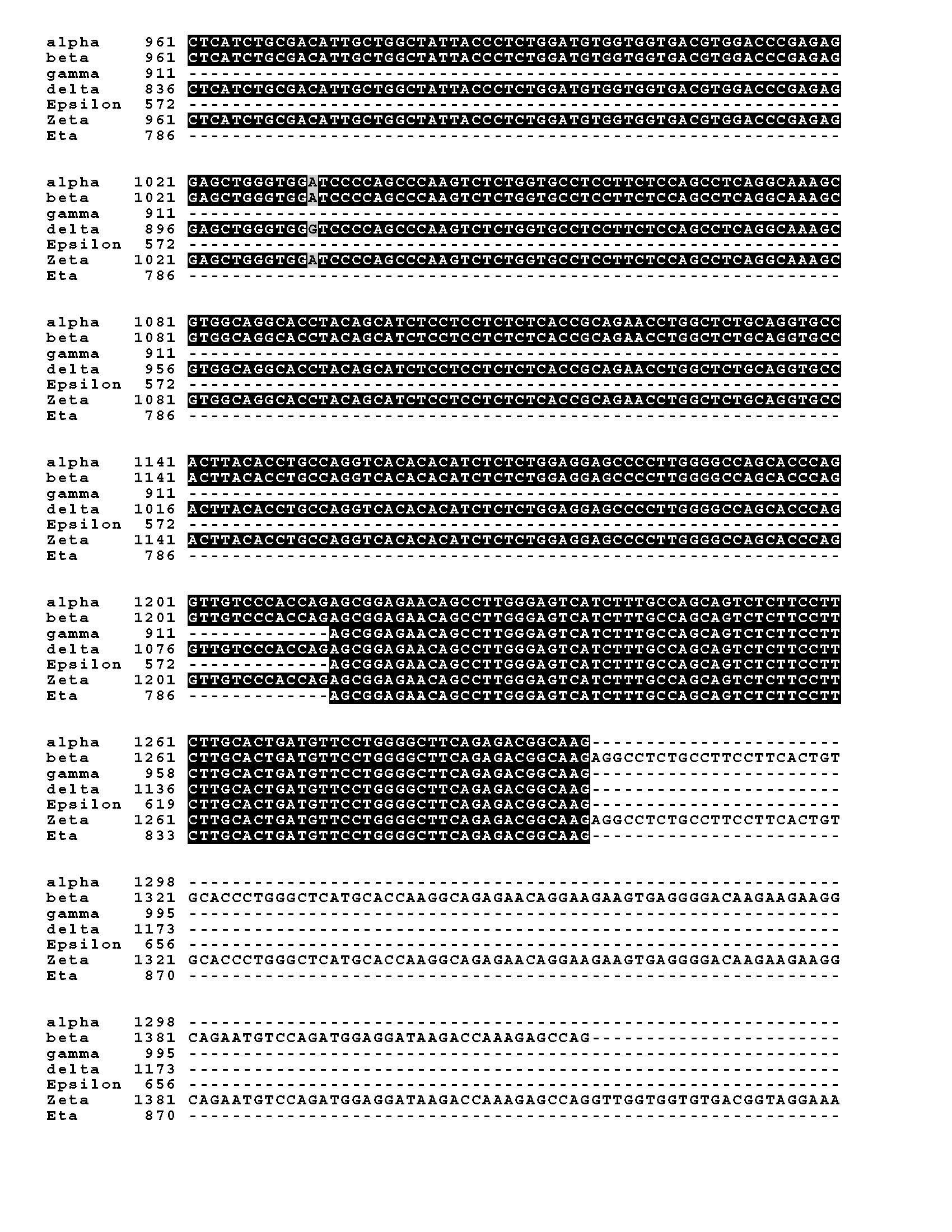

Supplement: Figure S3 — Expression of the TAPBPR β and γ transcripts in human tissue. [file imm0142-0289-sd3.tiff]

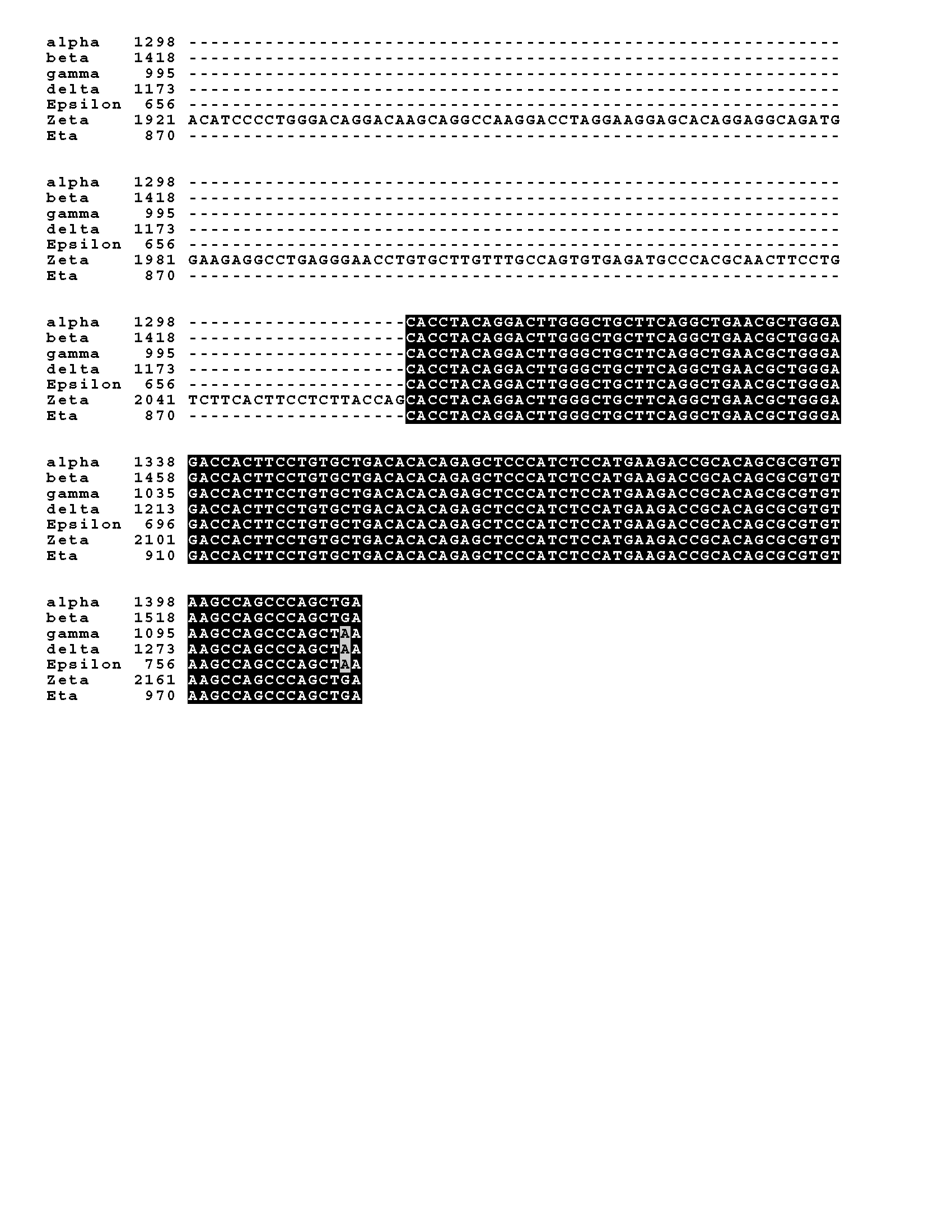

Supplement: Supplementary file 5 [file imm0142-0289-sd5.tiff]
